# Exploring Contactless Vital Signs Collection in Video Telehealth Visits Among Veterans Affairs Providers and Patients: Pilot Usability Study

**DOI:** 10.2196/60491

**Published:** 2024-10-23

**Authors:** Lynn Garvin, Eric Richardson, Leonie Heyworth, D Keith McInnes

**Affiliations:** 1 Center for Healthcare Optimization and Implementation Research Veterans Affairs Boston Healthcare System Boston, MA United States; 2 Department of Health Law, Policy and Management Boston University School of Public Health Boston, MA United States; 3 Office of Connected Care/Telehealth US Department of Veterans Affairs Central Office Washington, DC United States; 4 Department of Medicine School of Medicine University of California, San Diego San Diego, CA United States; 5 Center for Healthcare Optimization and Implementation Research Veterans Affairs Bedford Healthcare System Bedford, MA United States

**Keywords:** veteran, provider, video-based care, vital statistics, telemonitoring, usability, mobile health app, telemedicine, health care access, vital sign, video, telehealth, patient, Veterans Affairs, telehealth platform, photoplethysmography, camera, web-based survey, electronic medical record, home-based biometric devices, mHealth

## Abstract

**Background:**

To expand veterans’ access to health care, the Veterans Affairs (VA) Office of Connected Care explored a novel software feature called “Vitals” on its VA Video Connect telehealth platform. Vitals uses contactless, video-based, remote photoplethysmography (rPPG) through the infrared camera on veterans’ smartphones (and other devices) to automatically scan their faces to provide real-time vital statistics on screen to both the provider and patient.

**Objective:**

This study aimed to assess VA clinical provider and veteran patient attitudes regarding the usability of Vitals.

**Methods:**

We conducted a mixed methods evaluation of Vitals among VA providers and patients, collecting data in July and August 2023 at the VA Boston Healthcare System and VA San Diego Healthcare System. We conducted analyses in October 2023. In-person usability testing sessions consisted of a think-aloud procedure while using the software, a semistructured interview, and a 26-item web-based survey.

**Results:**

Usability test sessions with 20 VA providers and 13 patients demonstrated that both groups found Vitals “useful” and “easy to use,” and they rated its usability highly (86 and 82 points, respectively, on a 100-point scale). Regarding acceptability or willingness/intent to use, providers and patients generally expressed confidence and trust in Vitals readings, with high ratings of 90 and 85 points, respectively. Providers and patients rated Vitals highly for its feasibility and appropriateness for context (90 and 90 points, respectively). Finally, providers noted that Vitals’ flexibility makes it appropriate and advantageous for implementation in a wide range of clinical contexts, particularly in specialty care. Providers believed that most clinical teams would readily integrate Vitals into their routine workflow because it saves time; delivers accurate, consistently collected vitals; and may reduce reporting errors. Providers and veterans suggested training and support materials that could improve Vitals adoption and implementation.

**Conclusions:**

While remote collection of vital readings has been described in the literature, this is one of the first accounts of testing a contactless vital signs measurement tool among providers and patients. If ongoing initiatives demonstrate accuracy in its readings, Vitals could enhance telemedicine by providing accurate and automatic reporting and recording of vitals; sending patients’ vital readings (pending provider approval) directly to their electronic medical record; saving provider and patient time; and potentially reducing necessity of some home-based biometric devices. Understanding usability issues before US Food and Drug Administration approval of Vitals and its implementation could contribute to a seamless introduction of Vitals to VA providers and patients.

## Introduction

Veterans’ access to high-quality health care is a priority for the US Department of Veterans Affairs (VA), which serves over 9 million enrolled veterans each year [1]. VA has built its telehealth services over the past 20 years, with 40% of veterans now receiving some portion of their VA care through telehealth [2]. During this time, VA and US policy makers have sought to address the digital divide faced by many veterans due to demographic, clinical, and geographic challenges (eg, travel distance to care in rural settings) [3,4]. In 2016, the VA’s Office of Rural Health and Office of Connected Care (OCC) launched a national initiative to distribute video-enabled tablets to veterans facing access barriers [5,6]. The VA Maintaining Internal Systems and Strengthening Integrated Outside Networks Act of 2018 (MISSION Act; Public Law 115-182) eliminated geographic licensure constraints, permitting VA providers to care for veterans across state lines in the United States, its possessions, and territories [7]. In 2019, VA partnered with mobile carriers to reduce data fees for veterans using VA video telehealth [8]. To meet the sudden demand for telemedicine posed by the COVID-19 pandemic, in 2020, VA’s video architecture was rapidly scaled to allow over 3000% growth in video telehealth in 1 year [6]. Simultaneously, OCC launched its Digital Divide Consult, which initiated tablet distribution to eligible veterans [9] and, in 2021, partnered with the US Department of Housing and Urban Development-VA Supportive Housing program to distribute smartphones and tablets to homeless veterans [10] to support their telemedicine access. Current expansion of VA video telehealth continues to focus on quality and equity [11,12].

One critical telemedicine solution is the VA Video Connect (VVC) secure videoconferencing app, designed to help veterans meet with their health care teams on any smartphone, computer, or tablet [13]. Studies have shown that video telehealth can offer effective delivery of mental health care [14-16], primary care [17,18], and specialty ambulatory care [19,20]. Patient populations who face demographic, clinical, and geographic challenges (eg, travel distance to care in rural settings) can benefit from video telehealth [3,4].

**Figure 1:**
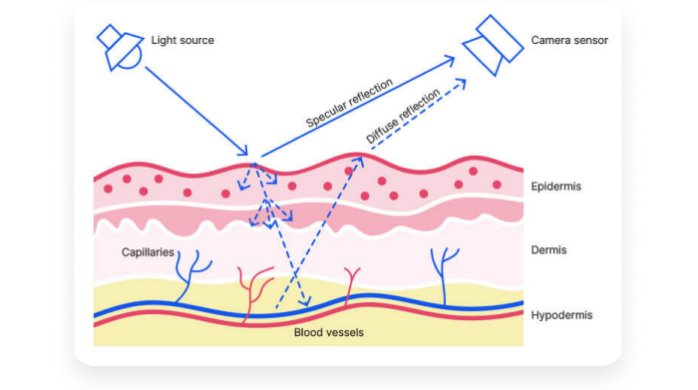
Remote photoplethysmography technology on smartphones can scan patient faces to deliver vital statistics for patients and providers to view.

The VA OCC, which manages VVC, is constantly exploring new innovations. A novel VVC software feature called “Vitals” uses contactless, video-based, remote photoplethysmography (rPPG) technology through the infrared camera on veterans’ smartphones (and other devices) to automatically scan their faces when incorporated into the video platform (Figure 1). Within 45 seconds, it delivers vital statistics on screen to both the provider and patient. Vitals’ statistics include blood pressure, respiratory and heart rates, pulse, and temperature.

The artificial intelligence–powered, video-based Vitals software was developed by Binah.ai, a health care technology company. To explain how Vitals is used in conjunction with the VVC videoconferencing app, a VA solutions provider, Iron Bow Technologies, developed a prototype app that integrates the Pexip video solution (the same one used for VVC) with the Binah platform (Android SDK) to allow Vitals to be displayed through a video call transmitted over the Pexip video. US Food and Drug Administration (FDA) approval for Vitals software is pending.

If Vitals demonstrates accuracy in its readings (as tested through a separate project), it could enhance telemedicine by providing accurate and automatic reporting and recording of vitals; sending patients’ vital readings (pending provider approval) directly to their electronic medical record, thus saving provider and patient time; and potentially reducing necessity of some home-based biometric devices. There are a number of current VA initiatives in which remote vital sign collection would be beneficial, such as in preanesthesia patient evaluations [21]. While remote collection of vital readings has been described in the literature [22], this is one of the first accounts of testing a contactless vitals tool among providers and patients.

Hence, the objective of this project was to evaluate VA provider and veteran attitudes regarding the usability of, and intent to use, Vitals. Related attitudes of trust, privacy, credibility, personal preference, and practice were explored. Providers and veterans (hereafter “patients”) might voice a range of interests, questions, or concerns regarding Vitals and its potential to support accurate and transparent health information collection, data sharing, and use. Understanding these issues before FDA approval and implementation could contribute to a seamless introduction of Vitals to VA providers and patients.

## Methods

### Overview

We conducted a mixed methods evaluation of VA provider and patient attitudes regarding the usability of Vitals and their intent to use. In-person usability testing sessions consisted of a think-aloud procedure and a semistructured interview, followed by a quantitative survey. We collected data in July and August 2023 and conducted analysis in October 2023.

### Participants

We recruited participants from the primary and specialty care programs of the VA Boston Healthcare System and VA San Diego Healthcare System. A prospective sample of 15 providers (7-8 per site) included 5 primary care and 10 specialty care providers. Based on studies of specialty care telemedicine [19,20,23] and guidance from OCC, 10 specialty care providers were selected from across specialties that could most benefit from Vitals, namely, dermatology, endocrinology (eg, type 1 and type 2 diabetes), oncology, gastroenterology, pulmonology (eg, chronic obstructive pulmonary disease), nephrology (eg, chronic kidney disease), cardiology (eg, congestive heart failure), neurology (eg, functional neurological disorder), anesthesiology, pain management, rehabilitation (eg, physical or speech therapy), and emergency care or urgent care. A prospective sample of 20 patients (10 per site) included 10 “less experienced” patients who had 1-2 VVC visits and 10 “more experienced” patients who had 3+ VVC visits within the past year on tablets (no phones or computers). The sample reflected a variety of health conditions relevant to vital statistics, for example, diabetes or chronic obstructive pulmonary disease, and provided a broad sociodemographic representation. Based on qualitative research standards, “data saturation” is the point at which no new information or themes are observed. We determined that saturation could be reached with 5-10 individual interviews per sample [24,25].

### Usability Testing Among Providers and Patients

We conducted 30-minute, in-person sessions with providers and 60-minute, in-person sessions with patients. Researchers LG and ER conducted sessions in Boston and researcher DM conducted sessions in San Diego. Sessions consisted of two steps: (1) usability testing through a think-aloud protocol and interview, and (2) a structured survey.

#### Step 1: Usability Testing and Semistructured Interviews

**Figure 2:**
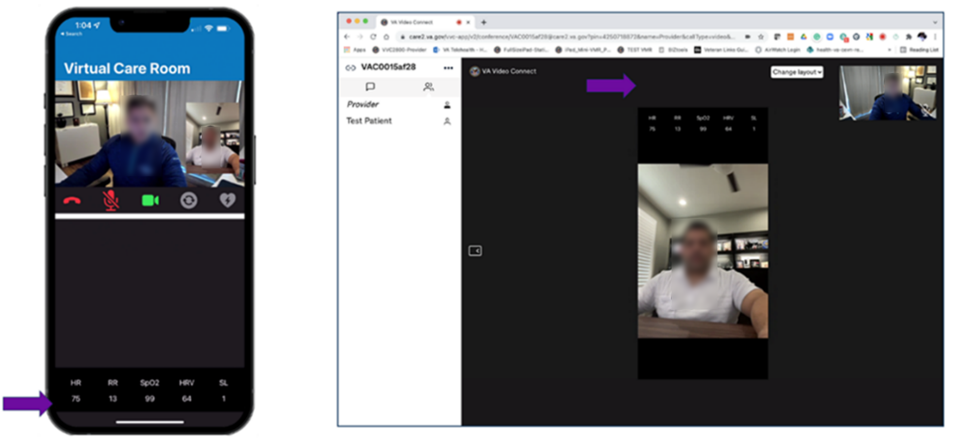
Views of Vitals on veterans’ smartphones and on VA providers’ tablets (the purple arrows point to the vital sign readings on both devices).

Usability testing combined 2 qualitative methods, a think-aloud procedure and an interview. In a think-aloud exercise, the participant is asked to voice their thoughts while examining or navigating the use of a technical device or software [26-28]. To make the think-aloud experience as real as possible, we conducted mock VVC visits among patients, with the researcher playing the role of the provider collecting the patient’s vital signs using Vitals. Patients were given Vitals-enabled smartphones while the researcher acting as the provider used a Vitals-enabled clinical tablet. Alternatively, the researcher played the role of the patient with VA providers. This procedure enabled us to gather participants’ real-time impressions while observing their facial cues and body language while they were using Vitals. Static visuals (storyboards, Figure 2) of the providers’ and patients’ views of Vitals on a tablet served as a backup tool for interviews in case issues arose that prevented the tablet, VVC, or Vitals from operating.

The semistructured interviews that followed documented participants’ attitudes about Vitals based on constructs of the Unified Theory of Acceptance and Use of Technology (UTAUT) [29] (detailed in the *Measurement and Instrument* subsection).

#### Step 2: Quantitative Survey

We concluded our session with the collection of provider and patient responses to a brief quantitative survey, composed of validated scales for intervention usability, acceptability (willingness to use), appropriateness (fit for purpose), and feasibility. Researchers read survey questions aloud to patients and entered responses to the web-based survey hosted on VA’s secure Research Electronic Data Capture (REDCap; Vanderbilt University) platform [30]. Patient participants received a US $25 CVS gift card as reimbursement for completing the session. Being familiar with REDCap, providers entered their own responses to the web-based survey.

### Measurement and Instrument

The semistructured interview guides for providers and patients were informed by the UTAUT model, which proposes that performance expectancy, effort expectancy, social influence, and facilitating conditions (positive and negative) influence a person’s intent to use, the strongest predictor of technology adoption [29]. Other themes explored included participants’ attitudes on Vitals’ measurement accuracy, data privacy, system security, and their trust in the VVC app. Interview questions asked whether any training, educational materials, or technical support were needed and probed for barriers and facilitators to Vitals’ acceptance and maintained use (eg, impact on clinical workflow). Table 1 summarizes the constructs, measures, and their sources (refer to Multimedia Appendices 1-4 for interview guides and web-based surveys for providers and patients).

The 26-item survey quantitative survey that concluded each session was composed of 4 validated scales: the Intervention Usability Scale (IUS) [31], the Acceptability of Intervention Measure (AIM) [34], the Feasibility of Intervention Measure (FIM) [34], and the Intervention Appropriateness Measure (IAM) [34]. All measures use Likert-style questions on a 5-point continuum. Based on research on the System Usability Scale [32,33] (the parent scale for IUS), a score above 68 points is considered above average and anything below 68 points is below average [32,35].

**Table 1:** Constructs, measures, and sources: usability evaluation.

Steps and constructs			Measures
(**Step 1a) Think-aloud protocol**			
	User perceptions	Unfiltered user impressions of the usability and acceptability of Vitals tool.	
**(Step 1b) Qualitative measures^a^**			
	Performance expectancy	Perceived effectiveness and usefulness, for example, provider time saved in recording accurate blood pressure reading; equivalent care quality for VVC^b^ visit as an in-person visit.	
	Effort expectancy	Design simplicity and ease of use, for example, patient convenience of not having to travel for care; upon provider approval, Vitals automatically adds the reading to patient health record.	
	Social influence	Community and provider support for use, for example, availability of technology and training.	
	Facilitating conditions	Facilitators or barriers to technology use, for example, friends who assist with technology is a positive facilitating condition, while depression can be a negative facilitating condition.	
**(Step 2) Quantitative measures^c^**			
	Usability	Factors that affect providers’ and patients’ ability to use the solution to achieve specified goals. The 10-item Intervention Usability Scale [31], appropriate for combined interpersonal and technology solutions, is an adaptation of the widely used System Usability Scale [32,33].	
	Acceptability	Factors that affect providers’ and patients’ willingness or intent to use a solution deemed enjoyable and comfortable to use; 4-item Acceptability of Intervention Measure [34].	
	Feasibility	Motivation and ability to introduce and support the solution, and the extent to which the solution is practical and can be successfully used or completed. Feasibility will be assessed with the 4-item Feasibility of Intervention Measure [34].	
	Appropriateness	Factors that affect the perceived fit (relevance and suitability) of the solution to a given context, a given provider or patient, or solving a particular issue or problem; 4-item Intervention Appropriateness Measure [34].	

^a^Qualitative measures: patient and provider usability interviews based on Unified Theory of Acceptance and Use of Technology constructs [29].

^b^VVC: VA Video Connect.

^c^Quantitative measures: survey administered at the conclusion of patient and provider interviews. All measures use Likert-style questions on a 5-point continuum [31-34].

### Data Analysis

For qualitative analysis, audio recordings of usability testing sessions were transcribed. These, along with session notes, were coded thematically using rapid qualitative inquiry method [36,37]. Researchers LG and ER coded interviews, performing inductive and deductive content analysis. A priori categories included UTAUT constructs (performance expectancy, effort expectancy, social influence, and facilitating conditions) and other themes from the literature (see the *Measurement and Instrument* section). Emergent themes from participants’ think-aloud comments generated additional codes. The research team conducted a thematic analysis that assessed the patterns of attitudes and experiences and reached consensus on interpretation.

For quantitative analysis, we calculated total scores for the survey measures following the published guidelines for scoring. For ease of interpretation, scores for the AIM, FIM, and IAM were converted to a 100-point scale to match the IUS scores. We then calculated descriptive statistics (eg, range and mean) of the total scores on measures. We calculated descriptive statistics of individual items to capture some additional insights into participant responses. Analysis was conducted using R statistical software (version 4.2; R Foundation for Statistical Computing) [38].

### Ethical Considerations

All procedures were approved and considered quality improvement by the Institutional Review Board at the VA Boston Health care System in March 2023. The Institutional Review Board of the VA Boston Healthcare System determined this protocol (1685680-1) “VA Video Connect Vitals – Evaluation of Usability and Intent to Use” to be exempt on May 25, 2022. All participants provided verbal informed consent for their participation and audio recording.

## Results

### Sample Characteristics

We interviewed 20 VA providers whose average age was 47 (range 30-63) years. Over half (12/20, 60%) identified as female. An equal number of providers identified their race as Asian (7/20, 35%) or White (7/20, 35%). Service line representation included primary care (9/20, 45%) and specialty care (5/20, 25%), with remaining indicating a telehealth specific position. Specialty care included urgent care, neurology physical therapy, and surgery. Their experience with telehealth was broadly distributed.

We also interviewed 13 patients with an average age of 67 (range 38-84) years, all of whom identified as male. Race and ethnicity were evenly divided among patients who self-identified as either Black or African American, Hispanic or Latinx, or Asian. All patients had attained at least a high school diploma. Most patients (12/13, 90%) lived 30 to 60 minutes from their VA facility.

### Usability of Vitals

**Figure 3:**
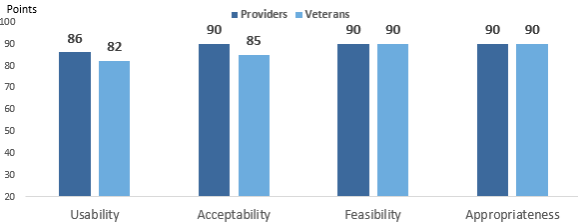
Veterans Affairs provider and veteran average score (out of 100 points) for Vitals on scales for usability, acceptability, feasibility, and appropriateness to context.

As seen in Figure 3, providers and patients highly rated Vitals’ usability (86 points and 82 points, respectively). Overall, providers thought that Vitals was “useful” and “easy to use.”

I like it. That’s my biggest thing with doing VVCs is sometimes not having vitals...One of the biggest things I see is hypertension, and the blood pressure is always elevated. And so that's like the biggest thing I would want to see is the blood pressure...

Provider 202, NP

I think -- it seems pretty easy...I think having batteries and making sure you find a finger if they have...circulation issues, that this [Vitals] is almost easier than using the pulse oximeter.

Provider 105, RN

Providers liked that Vitals “helps to provide some valuable context” and “helps to triage” patient’s general health and particular conditions.

I do actually like the stress level. That’s something we don’t do in person. We ask Veterans manually about what their stress levels are...So, more of an objective look at stress levels...So, that’s one I think that’s in here that I don’t think we get normally and I would like.

Provider 211, MD

Patients found Vitals both easy to use and more convenient than conventional methods of reading vitals remotely, for example, using a blood pressure cuff or oxygen sensor.

It’s a lot easier to sit still and relax in front of a computer screen monitor than it is holding your phone.

Veteran 201

Well, right now you have to do a BP cuff and an O2 sensor, my scale, and then my finger for my glucose to get all the readings to provide...

Veteran 201

While Vitals usability scored high overall, providers rated 2 items on the IUS slightly lower, indicating the need for technical assistance at the introduction of the Vitals and evidence of Vitals’ accuracy based on its validity (measurement to standard) and reliability (consistently valid measurement over time). During the structured interviews, providers also emphasized that educational materials distributed to both providers and patients before use would be critical to effective use. Examples included a Vitals data sheet, Vitals onscreen prompts on patients’ smart devices to guide them through use experience, and video training on both the VA website and social media platforms, for example, YouTube (Google).

For providers in general, I’d probably start with evidence base...Like I would say “We can get this data and here is the basis of how this data is collected...Here is what the system, the software is doing to give us this data and here’s how it’s been validated.”

Provider 211, MD

[Need to provide patients with a prompt on screen before Vitals starts]...Maybe some sort of automated technical cue] “We are now collecting your vitals, so...Please make sure your face...stays centered”...or whatever you need to do to make sure you get the appropriate readings.

Provider 211, MD

Patients did not express any concerns with usability of Vitals. However, several patients mentioned the challenge of logging in or maintaining an internet connection throughout the visit. Furthermore, some patients mentioned that it would be challenging to hold up their smartphone or tablet for the required 45-60 seconds (to obtain the vitals reading) and suggested that VA provide a tabletop stand to hold and stabilize the device during the reading.

I think if -- releasing it [Vitals] with the blood pressure, you get way more utilization with it.

Veteran 201

...my thought just being that if you want consistency between the readings, there’s actually three variables. The head, the camera, and the hand. So, if you can remove variability for the camera and the hand, now it’s just the head.

Veteran 201

Even just something like a clipboard tripod. You know the old things that the secretaries would use...That simple stand somebody used to put a steno pad in and do their notes on.

Veteran 203

### Acceptability of Vitals

Providers and patients rated Vitals high on the AIM, reflecting their willingness to use Vitals (Figure 3). They perceived Vitals readings as being accurate, resulting in trust. Neither group expressed concern with security. Providers liked the fact that patients could take their own vitals anytime with the app and that it educates and engages them in their health, an important part of patient-centered care.

I feel like I could trust it. Yeah. To me it's all cool technology. Other big tech companies are doing this stuff, so I'm sure there is good evidence and research behind the technology. Yeah, I trust it.

Provider 201, DPN

I like the fact that the Veterans can see it themselves, too, which is helpful because they can get a little patient education with it, learn every time they do a VVC, they can see it.

Provider 206, Licensed Vocational Nurse (LVN)

I would at least be open [to] it, like if there was an issue with blood pressure or something like that, because a lot of times a lot of our Veterans have hypertension...so I know accurate readings are important...I think she’s [primary care physician] more in fear of ‘What if it’s inaccurate and I have to treat it and something happens to the patient.’ And it was – ‘Did it need to be treated.’ Or maybe ‘I didn’t treat something because it should’ve been treated.’ I think that’s more of a concern...

Provider 206, LVN

Patients felt that Vitals was acceptable for their care stating that it helped them keep track of their vitals between visits, was easier than other ways of collecting vitals, and helped with their telemedicine visits. They did not, however, want this to replace the option of seeing a doctor in person. One veteran expressed this qualified acceptance, saying the following:

...as long as the provider didn’t default and decline to see us when we felt like we needed to have an in-person visit.

Veteran 201

Patients offered suggestions on how to promote the technology to them, such as having providers explain Vitals’ value in collecting patient metrics, and VA offering materials on how Vitals’ readings compare with familiar consumer monitoring devices they use, for example, Google Fitbit and Apple Watch.

[Need for providers to offer some initial explanation of Vitals’ value to patients.] If you just give me data, I’m either going to be pissed off or I’m going to -- it’s going to usurp my confidence in the app, or both. And if it wasn’t important, they wouldn’t be measuring it.

Veteran 203

It was very close to heart rate [comparing Vitals to his Fitbit reading]. This had 97, that was one [sic] 98, so it’s very close. I don’t know how accurate this is sometimes...it seemed pretty accurate.

Veteran 203

### Feasibility of Vitals

In both the surveys and interviews, providers and patients found Vitals highly feasible and practical for home-based use. One patient expressed that Vitals could also be empowering (Figure 1).

I think it’s pretty cool. I think it would be nice because we don’t -- unless the patient has a blood pressure machine at home, we -- and they can’t -- they have to visually show us checking it, we can’t technically count it...So, it’d be nice because not everyone has a blood pressure cuff at home.”

Provider 206, LVN

It seems pretty cool. I like the technology to be able to do that at home instead of having to come in here. Like if I’m having a panic attack, it would be nice to see what’s going on with my heart rate and everything. Is the heart actually rapid or was it just a panic attack...‘cause it does feel like a heart attack sometimes [laughing].

Veteran 201

### Appropriateness of Vitals

Finally, providers and patients rated Vitals high on appropriateness, that is, fit for purpose, per the IAM (Figure 1). Providers believed that most clinical teams would readily integrate Vitals into their routine workflow because it saves time and delivers accurate, consistently collected vitals, and may reduce reporting errors in the electronic health record.

You could just say like ‘This has been validated and however it’s been validated’...then maybe references for people to look up. So, if I am a clinician who maybe is not necessarily believing and I wanna look at factual...strength of evidence...I can’t state that enough that it’s reproducible, valid, and correlates with what I would get if I had taken vital signs in person...knowing that both have problems.

Provider 211, MD

I want integration into CPRS,* and I want to make sure that it'll work with Genesis...

Provider 202, NP

VA’s Computerized Patient Record System (CPRS) is being replaced by the Cerner Millennium solution that powers the Department of Defense’s Military Health System (MHS GENESIS) [39].

Furthermore, providers from different specialties indicated that Vitals is appropriate in a variety of clinical contexts. For example, several specialty care providers mentioned how Vitals would enhance their workflow, help with billing for video-based visits, and keep patients safer for preparation appointments. Specialty providers said Vitals could be used for video-based physical therapy appointments and medication management with the pharmacy.

So, we could finish our exercise, we could say hit the heart button and then their vitals would be -- just be popping up as we go. Or I mean every [inaudible]. It would simplify what we do quite a bit.

Provider 203, DPN

“I know that with pharmacy, they see Veterans for hypertension to monitor their blood pressure so they can taper or increase or decrease their medication. So, I mean I don’t know how far it’s gonna go or who’s gonna access it, but I know it could be beneficial to like many people, not just primary care, but I know like there’s a lot of people who follow-up on this data...”

Provider 206, LVN

Also, primary care providers expressed that Vitals could help improve video-based visits and help streamline the collection of patient vitals. They also expressed interest in an application for follow-up visits.

I think we're trying to move towards check-ins, VVC check-ins, like, mirroring how it happens in real life. Like, when you go to a doctor, LVN or nurse comes and checks you in, takes your vitals. So, we're trying to mirror that or get to a point where we can mirror that with our virtual appointments, where an LVN will be checking them in. So, I think at that point would be a good point to get these vitals.

Provider DPN]

Yes. It's vastly [better] because there are no vitals. If we have any, it's because we're looking for the Veterans to provide those. You don't know if their instruments are up to date. I always ask, “How old is your blood pressure cuff?”...And you also wonder if they're coming to the provider, they have to give a good blood pressure reading...you want to be able to see something and to do it in real time, I think that's awesome.

Provider 201, LPN

We have a lot of screening questions. So, if it would be -- it’s perfect if it works and they can talk and we can continue through like the check-in process, because that’s when we would be using in.

Provider 206, LVN

## Discussion

### Principal Findings

Overall, health care providers and veteran patients at 2 VA facilities found that contactless remote collection of vital signs presents a usable, acceptable, feasible, and appropriate way to collect patient health information. Our findings highlight some of the similarities and unique perspectives concerning usability and intent to use among health care providers and patients. The inclusion of patients presents a full description of usability of contactless collection of vital signs, which is critical for developing implementation plans. This is one of the first accounts of testing a video-based vitals tool with patients.

First, providers and patients expressed strong support for Vitals’ usability in their discussion and survey scores. Through hands-on experience in a Vitals demonstration, they found the app to be useful and easy to use. Providers liked that Vitals “helps to provide some valuable context” and “helps to triage” patient’s general health and particular conditions. For successful adoption of Vitals, however, providers indicated their need for technical assistance during the Vitals’ introduction. Providers and patients emphasized that educational materials distributed before use would be critical to effective use. Providers would need a detailed report of Vitals’ accuracy based on its validity (measurement to standard) and reliability (consistently valid measurement over time) with references to peer-reviewed articles. Patients would need a clear, concise fact sheet. Providers and patients indicated that they would also benefit from video trainings (at VA provider- and patient-facing websites or YouTube). Providers suggested that patients’ ease of use could be increased if Vitals displayed onscreen prompts on patients’ digital devices (eg, to guide the patient through the Vitals process and display). Patients found Vitals more convenient than conventional remote monitoring because it required less equipment and setup. However, patients cautioned that VA and providers should make clear that they always have the choice of an in-person visit rather than a video-based visit. Providers and patients recognized that use of Vitals could eliminate the cost and inconvenience of some home-based peripherals (eg, blood pressure cuff) and potentially reduce the risk of patients’ misreading their vitals. Patients and providers urged support for veteran patients with mobility challenges (eg, hand tremors and loss of an arm), suggesting that VA offer a platform to steady the patient’s tablet or phone for accurate and consistent readings.

Second, regarding acceptability or willingness to use, providers generally expressed confidence and trust in Vitals’ readings. Still, they requested technical evidence, including citations to academic studies, to assure Vitals’ accuracy. Providers felt that use of Vitals would save time, potentially increase the accuracy of Vitals’ records, and promote more consistent clinical practice in taking vitals at the start of all visits. Patients also found Vitals acceptable and offered suggestions on how to present the technology to make it more acceptable to them, such as training providers to explain the Vitals app and its metrics during its introduction to patients, offering a device stand to steady the cameras of patients’ devices, and encouraging patients to compare Vitals’ readings with those of their Fitbit or Apple Watch.

Finally, providers noted that Vitals’ flexibility makes it feasible, appropriate, and advantageous for implementation in a wide range of clinical contexts, particularly in specialty care. Providers and patients both gave Vitals high marks for its feasibility and were pleased that it could eliminate the use of in-home peripherals and allow patients to take their vitals at any time. Providers believed that most clinical teams would readily integrate Vitals into their routine workflow because it saves time, delivers accurate and consistently collected vitals, and may reduce reporting errors. Providers signaled their readiness to implement Vitals once FDA approval was given and anticipated potential future capabilities. Specialty care providers, in particular, elaborated on how Vitals would enhance their workflow, help with billing for video-based visits, and keep patients safer for preparation appointments. Primary care providers expressed that Vitals could improve video-based visits and standardize vitals collection.

### Limitations

Several limitations to our findings should be considered. The small size of both provider and patient samples may have introduced potential bias. For example, all 13 patient participants were male, and they self-identified as either Black or African American, Hispanic or Latinx, or Asian. Offering some balance in representation, over half of providers identified as female and providers identified across all race and ethnicity categories. Participants were associated with only 2 VA health care systems, both in urban settings that precluded a rural patient or provider perspective. Future work should use larger, more geographically diverse samples of Vitals users, both providers and patients, as well as investigate the experiences of those who tried Vitals once but did not return, to understand why and how to promote ongoing use.

### Conclusions

An essential step in VA’s expanded access for veterans is the increase in usefulness and adoption of telemedicine tools such as VVC [11]. The contactless Vitals feature on their smart devices has the potential to make vitals collection faster and easier for VA providers and patients, and provide accurate, real-time vitals reporting and recording. Integration of this feature would first require FDA approval and technical development but offers the potential to support additional clinical use cases, especially for veterans with reduced mobility, those in underserved communities, and those in geographically remote areas to complete appointments when in-person care is not required. This ensures greater access to health care including treatment and prescribed medications and greater health care equity.

## Appendix

Multimedia Appendix 1 Semistructured interview guide for providers.

Multimedia Appendix 2 Semistructured interview guide for patients.

Multimedia Appendix 3 Web-based survey for providers.

Multimedia Appendix 4 Web-based survey for patients.

Multimedia Appendix 5 Equator Network SQUIRE checklist for quality improvement studies.

## Abbreviations

AIM: Acceptability of Intervention Measure
CPRS: computerized patient record system
FDA: US Food and Drug Administration
FIM: Feasibility of Intervention Measure
IAM: Intervention Appropriateness Measure
IUS: Intervention Usability Scale
LVN: Licensed Vocational Nurse
MHS: military health system
MISSION Act: Maintaining Internal Systems and Strengthening Integrated Outside Networks Act of 2014
OCC: VA Office of Connected Care
REDCap: Research Electronic Data Capture
rPPG: remote photoplethysmography
UTAUT: Unified Theory of Acceptance and Use of Technology
VA: US Department of Veterans Affairs
VC CORE: VA Virtual Care Consortium of Research
VVC: VA Video Connect

## Data Availability

Due to US Department of Veterans Affairs regulations and our ethics agreements, the analytic datasets used for this study are not permitted to leave the Veterans Affairs (VA) firewall without a data use agreement. This limitation is consistent with other studies based on VA data. However, VA data are made freely available to researchers with an approved VA study protocol. For more information, please contact the VA Information Resource Center at VIReC@va.gov.
