# Supplementary material for: Exploring Contactless Vital Signs Collection in Video Telehealth Visits Among Veterans Affairs Providers and Patients: Pilot Usability Study
Source: JMIR Form Res. 2024 Oct 23;8:e60491. doi: 10.2196/60491 (PMC11541150; doi:10.2196/60491)

***VA Office of Connected Care Vitals Usability***

***30-min Provider In-Person Interview Guide and Personal Questions***

***VVC Background Information for Researchers***

*Legend*

- *Italicized text serves as a guide to Researchers, not to be read aloud to Participants.*
- Regular text will be read by Researchers to Participants.

*Equipment to Bring to Interviews*

| *1. VA Laptop*  *2. VA Audio Recorder* | *3. VA Provider iPad*  *4. VA Patient iPad* |
| --- | --- |

*Links to Initiate Provider and Patient Demonstrations*

*Use the “Facility Static Link Generator” to create URLs and Alias/PINs for the test devices to be used for collecting vitals during the POC calls. Or Keith and Eric can replace Lynn’s Patient iPad Serial # (PVTF9FDX4YBQ1KV) in URL (below) with their iPad Serial #:*

[*https://care.va.gov/vvcapp/?name=VAStaff&join=1&media=1&escalate=1&conference=PVTF9FDX4YBQ1KV@care.va.gov&pin=15789348#*](https://care.va.gov/vvcapp/?name=VAStaff&join=1&media=1&escalate=1&conference=PVTF9FDX4YBQ1KV@care.va.gov&pin=15789348)

*Lynn Garvin Serial Number: PVTF9FDX4YBQ1KV*

*Keith McInnes Serial Number: F9FDX4hrq1kv*

*Eric Richardson Serial Number: F9FF11A2Q1KV*

*RedCap Survey on Your Laptop*

*Provider:* [*https://varedcap.rcp.vaec.va.gov/redcap/surveys/?s=89DNAAKL788YML9D*](https://varedcap.rcp.vaec.va.gov/redcap/surveys/?s=89DNAAKL788YML9D)

*Patient:* [*https://varedcap.rcp.vaec.va.gov/redcap/surveys/?s=MXPT8HCAFFPE7TXJ*](https://gcc02.safelinks.protection.outlook.com/?url=https%3A%2F%2Fvaredcap.rcp.vaec.va.gov%2Fredcap%2Fsurveys%2F%3Fs%3DMXPT8HCAFFPE7TXJ&data=05%7C01%7C%7C22fecacdfc9646e013a508db68db8251%7Ce95f1b23abaf45ee821db7ab251ab3bf%7C0%7C0%7C638219064210329050%7CUnknown%7CTWFpbGZsb3d8eyJWIjoiMC4wLjAwMDAiLCJQIjoiV2luMzIiLCJBTiI6Ik1haWwiLCJXVCI6Mn0%3D%7C3000%7C%7C%7C&sdata=EoTKyMi%2FX%2FrIfHpq4HekMjbrgA8D3ADd2Mpga150DD0%3D&reserved=0)

*Appendix*

*All Potential Vital Signs (for probe with Participants on other signs they would like to see)*

*Screenshot of Vitals (backup if online version is not working)*

*Vitals Descriptions and Abbreviations*

*Accuracy table and Report times includes more information on supported Vital Signs*

- *Heart Rate (****HR****) – The number of times a person’s heart beats per minute (BPM).*
- *Respiration Rate (****RR****) – Also referred to as “Breathing rate” is the number of breaths you take per minute.*
- *Oxygen Saturation (****SpO2****) – indicates the amount of oxygen traveling through your body with your red blood cells [presented as a percentage].*
- *Heart Rate Variability (****HRV****) – is the fluctuation in the time intervals between adjacent heartbeats. HRV indexes neuro-cardiac function and is generated by heart-brain interactions and dynamic non-linear autonomic nervous system processes.*
- *Stress Level (****SL****) – Binah’s Stress level is based on Baevsky’s and US/European Index level measurements (Globally approved) and is calculated according to Heart Rate Variability (HRV) measurements.*
- *Hemoglobin A1C (****HbA1c****) – represents the average blood glucose (sugar) level for the last two to three months. HbA1c is measured in percentage with resolution up to 0.01%.*
- *Blood Pressure (****BP****) – Also available in Binah Vitals, though not shown in this prototype.*
- *Vitals is in the process of receiving FDA approval but has not yet been FDA approved.*

***VA Video Connect Vitals***

***Provider In-Person Interview Guide and Online Survey***

***Verbal Consent, Permission to Record [2 mins]***

Good morning/afternoon. Thank you for meeting today for this 30-minute interview and survey. We will be exploring a new potential feature of the VA Video Connect telehealth application.

Your participation in this project is completely voluntary and you may choose to skip any questions you do not wish to answer or end the interview discussion at any time.

With your permission, we would like to audio-record this interview and survey so that we can reflect what you share with us accurately. All efforts will be made to protect your confidentiality. The audio recording of this interview will be stored on a secure VA server accessible only to the project team, using unique codes for each participant. The recordings will be transcribed and any potentially identifying information will be removed. We can pause the recorder during the interview if there is anything you do not want us to record.

Would it be alright to audio-record your interview and survey?

*If yes, continue with recording.*

*If no, continue without recording.* “Say, that’s alright, we won’t record this interview, I will take notes.”

***If verbal consent is given, turn on Audio Recorder. [If not, skip to next page]***

The audio recorder is on. Can you please confirm that we’ve reviewed the project information and that it is alright for us to record?

*[Participant confirmation]*

Thank you.

***Topic I: Provider Professional Characteristics [1 min]***

To start, we would like to ask a few questions about your practice:

1.1. Can you please describe the department or service line where you currently work?

Dept or Service Line: _______________________________________________________

1.2 What was the primary focus of your training and credentials received?

Credentials/Training: _______________________________________________________

(e.g., MD, DO, PhD, PsyD, DNP, PA, ANP, NP, RN et al.)

1.3. What proportion (%) of your appointments are currently virtual/telemedicine?

- Less than 10%
- 10-35%
- 35-64%
- 65-90%
- More than 90%

***Topic II: Vitals Demonstration and Think Aloud [5 mins]***

***Introduction to Evaluation***

Today, we are talking about your perceptions and intentions to use a new feature on VA Video Connect (VVC) called Vitals. Vitals uses the infrared camera on patients’ smartphones (and other devices) to automatically scan their faces during a video visit. (This is the same technology used for facial recognition, such as to unlock your phone when it recognizes you.) Within 45 seconds, it delivers vital statistics on screen to both the provider and patient. VVC Vitals statistics include: heart rate, heart rate variability, respiration rate, oxygen saturation, stress level, and hemoglobin A1C (blood glucose level).

Vitals can enhance virtual care by providing accurate, real-time reporting of vital signs to the provider and patient, and potentially reduce necessity for some home-based biometric devices (e.g., blood pressure cuff).

***Begin the demonstration by handing iPad to the participant, showing the Vitals screen***

Now, we will demonstrate the Vitals feature by simulating a telehealth appointment.

As you use the feature, please “think aloud” to tell us of your impressions and experiences.

Please note that the VVC screen in the demo may look a bit different from what you’re used to seeing. Patients see a similar readout on their screens.

*Questions to ask:*

2. What is your first reaction to seeing the Vitals readout screen?

3. Is there anything that stands out? Or anything missing?

4. How does this differ from your usual telehealth appointments? Which medical team member typically takes patient vitals during telehealth? Does this enhance or hinder?

5. How might Vitals fit into your particular practice?

The current Vitals statistics include: heart rate, heart rate variability, respiration rate, oxygen saturation, stress level, and hemoglobin A1C (blood glucose level).

6.1 Would these be helpful measures in your virtual visits? Keep them all? Drop any?

6.2 Are there other statistics that you would like to see? [List of Potential Statistics in Appendix]

Thank you for your input about the Vitals feature!

***Topic III: Effectiveness/usefulness, including trust question; validity question; security of data question; privacy of data question; etc. [5 mins]***

Next, we would like to ask a few questions about your perceptions of the accuracy, effectiveness, and security of VVC Vitals. The accuracy of a feature like Vitals is based on its validity (measurement to standard) and its reliability (provides consistently valid measurement over time).

7. Can you describe your perceptions of the accuracy of the Vitals reading provided by VVC Vitals?

***Probing questions****.* On a scale of 1 to 10 how accurate do you think the results are? What kept that number from being higher or lower?

8. How would you describe the usefulness of an application like VVC Vitals?

8.1. How would you compare the usefulness to in-person visits?

8.2 Would you imagine that using VVC vitals could speed/facilitate you/your patient’s sharing of information? (e.g., more frequent updates, reduce patient visits to clinic)

9. How would you describe your level of trust in using VVC Vitals?

9.1.Would you feel confident in reporting the results to patients? How so?

10. What are your thoughts on the privacy of data and security of data collected through Vitals?

***Probing questions****:* Describe your comfort level in recommending VVC Vitals to patients using telemedicine?

***Topic IV: Ease of use/convenience [5 mins]***

Now, we would like to ask some questions about the experience of using VVC Vitals.

11. How would you describe the experience of using VVC Vitals?

11. 1. Did you find the application easy to use? If yes, what was easy about the use? Or what was challenging about using Vitals? [What would be important elements of ease of use for use?]

11.2. How does using VVC Vitals compare to in-person vitals? [How do you think this VVC app would compare to your current in-person vitals collection?]

12. How would you describe the effort needed to use VVC Vitals? How does this compare with in person visits?

***Topic V:***  ***Vitals training and educational materials, support [3 mins]***

Next, we have a few questions about ways the VA can support the use of VVC Vitals.

13. What ways can the VA support your use of VVC Vitals?

13.1. What type of training should be offered to support your use or your team’s use of VVC Vitals?

13.2. What instructional materials would be helpful to you or your team?

14. What types of informational materials would be useful to provide to patients? What materials would help providers to introduce the Vitals feature to patients?

***Probing question****:* Do you think providers would use a brief script to help introduce the Vitals feature to patients?

15. Would the use of Vitals by your colleagues influence your decision to use Vitals? What role would the opinion of other professionals play in your decision?

***Topic VI:*** ***Other barriers and facilitators to Vitals’ acceptance and sustained use (e.g., clinical workflow) [4 mins]***

The next couple of questions ask about barriers to using VVC Vitals.

16. Can you please describe any barriers you see hindering use of VVC Vitals?

16.1. Are there any hesitations? If so, how would you describe any hesitations?

17. How would you describe VVC Vitals integration into workflow?

17.1. Does it enhance workflow? If so, how?

17.2 Does it disrupt workflow? If so, in what ways?

***Topic VII: Vitals RedCap Survey (Online) [3 mins]***

*Bring laptop with RedCap survey to Interviews. Allow Providers to enter their responses independently. If instead Provider wishes Researcher to read the questions aloud, then DO read aloud the introductory phrase for each section, then read each question, marking the participant’s response.*

Provider: <https://varedcap.rcp.vaec.va.gov/redcap/surveys/?s=89DNAAKL788YML9D>

***Topic VIII: Conclusion and Provider Personal Characteristics [2 mins]***

To conclude, we would like to ask a few demographic questions.

18.1. What is your race/ethnicity? You may select more than one.

- Black or African American (Examples: African American, Jamaican, Ethiopian, Haitian, etc.)
- American Indian or Alaska Native (Examples: Navajo Nation, Blackfeet Tribe, Mayan, Aztec, etc.)
- Asian (Examples: Chinese, Vietnamese, Indian, Lebanese, etc.)
- Native Hawaiian or Pacific Islander (Examples: Kanaka Maoli, Samoan, Chamorro, etc.)
- Hispanic, Latinx, or Spanish Origin (Examples: Puerto Rican, Cuban, Salvadoran, etc.)
- White (of European, Middle Eastern or North African descent, Examples: Irish, German, Italian, Lebanese, Arab, Moroccan or Caucasian)

18.2. What is your gender?

- Female
- Male
- Transgender
- Do not identify as male or female
- Decline to answer

Thank you for taking the time to share your experiences on Vitals with me today.

We appreciate your contribution to our project. [Turn off audio recorder]

**APPENDIX**

***All Potential Vital Signs to Prompt Participants [Only 6 Currently Available]***


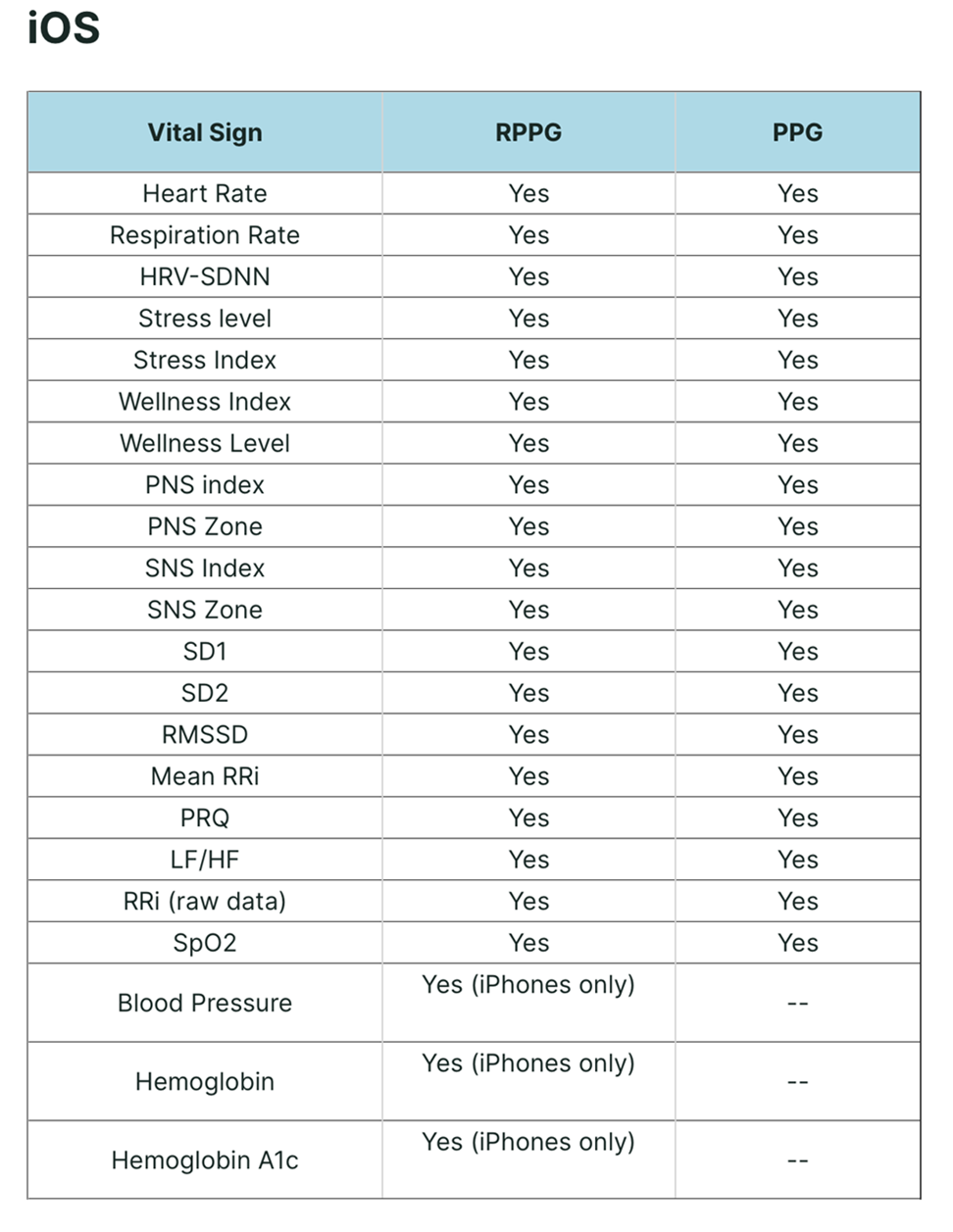


***Backup if Vitals Online is Not Operational: Think Aloud Procedure [3 mins]***

This screenshot shows what the Vitals reading looks like on your screen. Patients see a similar readout on their screens. Please note that the VVC screen in the demo may look a bit different from what you’re used to seeing.


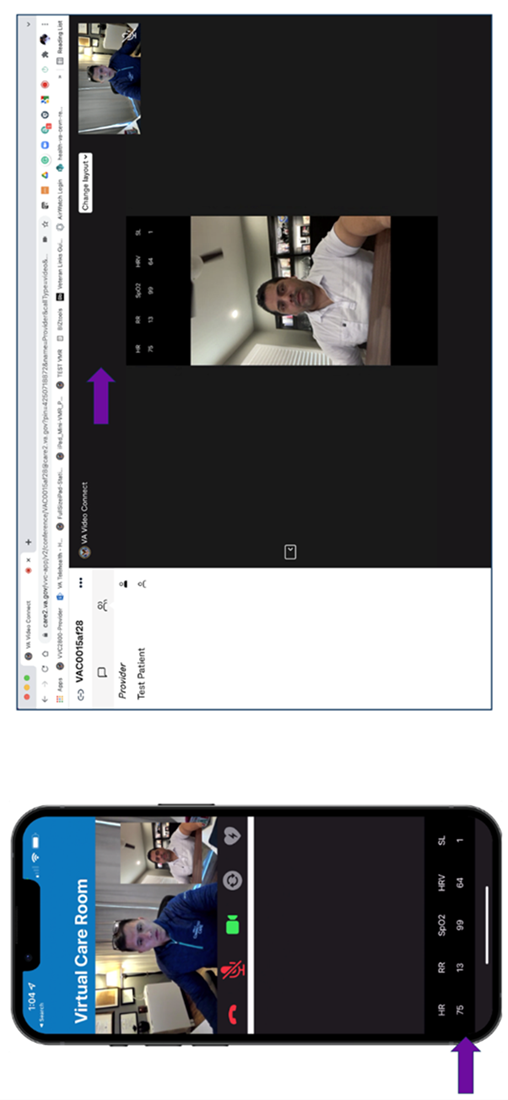

Supplement: Multimedia Appendix 1 [file formative_v8i1e60491_app1.docx]
