# Supplementary material for: Exploring Contactless Vital Signs Collection in Video Telehealth Visits Among Veterans Affairs Providers and Patients: Pilot Usability Study
Source: JMIR Form Res. 2024 Oct 23;8:e60491. doi: 10.2196/60491 (PMC11541150; doi:10.2196/60491)

***VA Office of Connected Care Vitals Usability***

***45-to-60-min Patient In-Person Interview Guide and Online Survey***

***VVC Background Information for Researchers***

*Legend*

- *Italicized text serves as a guide to Researchers, not to be read aloud to Participants.*
- Regular text will be read by Researchers to Participants.

*Equipment to Bring to Interviews*

| *1. VA Laptop*  *2. VA Audio Recorder* | *3. VA Provider iPad*  *4. VA Patient iPad* |
| --- | --- |

*Links to Initiate Provider and Patient Demonstrations*

*Use the “Facility Static Link Generator” to create URLs and Alias/PINs for the test devices to be used for collecting vitals during the POC calls. Or Keith and Eric can replace Lynn’s Patient iPad Serial # (PVTF9FDX4YBQ1KV) in URL (below) with their iPad Serial #:*

[*https://care.va.gov/vvcapp/?name=VAStaff&join=1&media=1&escalate=1&conference=PVTF9FDX4YBQ1KV@care.va.gov&pin=15789348#*](https://care.va.gov/vvcapp/?name=VAStaff&join=1&media=1&escalate=1&conference=PVTF9FDX4YBQ1KV@care.va.gov&pin=15789348)

*Lynn Garvin Serial Number: PVTF9FDX4YBQ1KV*

*Keith McInnes Serial Number: F9FDX4hrq1kv*

*Eric Richardson Serial Number: F9FF11A2Q1KV*

*RedCap Survey on Your Laptop*

*Provider:* [*https://varedcap.rcp.vaec.va.gov/redcap/surveys/?s=89DNAAKL788YML9D*](https://varedcap.rcp.vaec.va.gov/redcap/surveys/?s=89DNAAKL788YML9D)

*Patient:* [*https://varedcap.rcp.vaec.va.gov/redcap/surveys/?s=MXPT8HCAFFPE7TXJ*](https://varedcap.rcp.vaec.va.gov/redcap/surveys/?s=MXPT8HCAFFPE7TXJ)

*Appendix*

*All Potential Vital Signs (for probe with Participants on other signs they would like to see)*

*Screenshot of Vitals (backup if online version is not working)*

*Vitals Descriptions and Abbreviations*

*Accuracy table and Report times includes more information on supported Vital Signs*

- *Heart Rate (****HR****) – The number of times a person’s heart beats per minute (BPM).*
- *Respiration Rate (****RR****) – Also referred to as “Breathing rate” is the number of breaths you take per minute.*
- *Oxygen Saturation (****SpO2****) – indicates the amount of oxygen traveling through your body with your red blood cells [presented as a percentage].*
- *Heart Rate Variability (****HRV****) – is the fluctuation in the time intervals between adjacent heartbeats. HRV indexes neuro-cardiac function and is generated by heart-brain interactions and dynamic non-linear autonomic nervous system processes.*
- *Stress Level (****SL****) – Binah’s Stress level is based on Baevsky’s and US/European Index level measurements (Globally approved) and is calculated according to Heart Rate Variability (HRV) measurements.*
- *Hemoglobin A1C (****HbA1c****) – represents the average blood glucose (sugar) level for the last two to three months. HbA1c is measured in percentage with resolution up to 0.01%.*
- *Blood Pressure (****BP****) – Also available in Binah Vitals, though not shown in this prototype.*
- *Vitals is in the process of receiving FDA approval but has not yet been FDA approved.*

***VA Video Connect Vitals Evaluation***

***Patient Interview Guide and Online Survey***

***Verbal Consent, Permission to Record [2 mins]***

Good morning/afternoon. Thank you for meeting today for this 45-60 minute interview and survey. We will be exploring a new potential feature of the VA Video Connect telehealth application.

Your participation in this project is completely voluntary and you may choose to skip any questions you do not wish to answer or end the interview discussion at any time.

With your permission, we would like to audio-record this interview and survey so that we can reflect what you share with us accurately. All efforts will be made to protect your confidentiality. The audio recording of this interview will be stored on a secure VA server accessible only to the research team, using unique codes for each participant. The recordings will be transcribed and any potentially identifying information will be removed. We can pause the recorder during the interview if there is anything you do not want us to record.

Would it be alright to audio-record your interview and survey?

If yes, continue with recording.

If no, continue without recording. “Say, that’s alright, we won’t record this interview, I will take notes.”

***If verbal consent is given, turn on Audio Recorder. [If not, skip to next page]***

The audio recorder is on. Can you please confirm that we’ve reviewed the project information and that it is alright for us to record this interview?

*[Participant confirmation]*

Thank you.

***Topic I: Patient Health Characteristics [1 min]***

To start, we would like to ask a few questions about you and your health:

1.1. In general, how would you rate your overall physical health?

- Excellent
- Very good
- Good
- Fair
- Poor

1.2. In general, how would you rate your overall mental or emotional health?

- Excellent
- Very good
- Good
- Fair
- Poor

1.3. What is your current age? ____ years

1.4. How much time does it take for you to travel to your nearest VA facility?

- Less than 30 minutes
- 30 minutes to 1 hour
- 1 hour to 1 ½ hours
- 1 ½ hours to 2 hours
- Over 2 hours

Thank you.

***Topic II: Vitals Demonstration and Think Aloud [10 mins]***

***Introduction to Evaluation***

Today, we are talking about your perceptions and intentions to use a new feature on VA Video Connect (VVC) called Vitals. Vitals uses the infrared camera on patients’ smartphones (and other devices) to automatically scan their faces during a video visit. (This is the same technology used for facial recognition, such as to unlock your phone when it recognizes you.) Within 45 seconds, it delivers vital statistics on screen to both the provider and patient. VVC Vitals statistics include: heart rate, heart rate variability, respiration rate, oxygen saturation, stress level, and hemoglobin A1C (blood glucose level).

Vitals can enhance virtual care by providing accurate, real-time reporting of vital signs to the provider and patient, and potentially reduce necessity for some home-based biometric devices (e.g., blood pressure cuff).

***Begin the demonstration by handing iPad to the participant, showing the Vitals screen***

Now, we will demonstrate the Vitals feature by simulating a telehealth appointment.

As you use the feature, please “think aloud” to tell us of your impressions and experiences.

Please note that the VVC screen in the demo may look a bit different from what you’re used to seeing. Providers see a similar readout on their screens.

*Questions to ask:*

2. What is your first reaction to seeing the Vitals readout screen?

3. Is there anything that stands out? Or anything missing?

4. Are you able to see the Vitals readings clearly? Should the readings be larger?

5. How does this differ from your usual telehealth appointments? Which medical team member (e.g., doctor, nurse, health tech) typically takes your vitals during a telehealth visit?

The current Vitals statistics include: heart rate, heart rate variability, respiration rate, oxygen saturation, stress level, and hemoglobin A1C (blood glucose level).

6.1 Would these be helpful measures in your virtual visits? Keep them all? Drop any?

6.2 Are there other statistics that you would like to see? [List of Potential Statistics in Appendix]

Thank you for your input about the Vitals feature!

**Topic Area III: Effectiveness/usefulness, including: trust question; validity question; security of data question; privacy of data question; etc. [5-10 mins]**

Next, we would like to ask a few questions about your perceptions of the accuracy, effectiveness, and security of VCC Vitals. The accuracy of a feature like Vitals is based on its validity (measurement to standard) and its reliability (provides consistently valid measurement over time).

7. Can you describe your perceptions of the accuracy of the Vitals reading provided by VCC Vitals?

**Probing questions.** On a scale of 1 to 10 how accurate do you think the results are? What kept that number from being higher or lower?

8. How would you describe the usefulness of an application like VCC Vitals?

8.1. How would you compare the usefulness to in-person visits?

9. How would you describe your level of trust in using VCC Vitals?

9.1. Would you feel confident in the results when your VA provider gives them to you? How so?

10. What are your thoughts on the privacy of data and security of data collected through VCC Vitals?

**Probing questions:** Describe your comfort level in using VCC Vitals during a telemedicine visit?

Comparing Vitals to in-person visits, how would you describe, if any, the difference in your sense of control over collecting your Vitals?

**Topic Area IV: Ease of use/convenience [5-10 mins]**

Now, we would like to ask some questions about the experience of using VCC Vitals.

11. How would you describe the experience of using VCC Vitals?

11.1. Did you find the application easy to use? If yes, what was easy about the use? Or what was challenging about using Vitals?

11.2. How does using VCC Vitals compare to in-person vitals?

12. How would you describe the effort needed to use VCC Vitals? How does this compare with your VA provider collecting your Vitals during an in-person visit?

**Topic Area V:**  **Vitals training and educational materials, support [5 mins]**

Next, we have a few questions about ways the VA can support the use of VCC Vitals.

13. As a patient, what ways can the VA support your use of VCC Vitals?

13.1. What specific ways could your VA provider support your use of VCC Vitals?

14. What informational materials would be helpful to facilitate Vitals use?

15. Would the endorsement of your VA provider influence your decision to use Vitals?

15.1. Would the approval of friends or family influence your decision to use Vitals?

15.2. What role would the opinion of a caregiver or others play in your decision?

**Topic Area VI:** **Other barriers and facilitators to Vitals’ acceptance and sustained use**

**[5-10 mins]**

The next couple of questions ask about barriers to using VCC Vitals.

16. Can you please describe any barriers you see hindering your use of VCC Vitals during a telemedicine visit?

16.1. Are there any hesitations? If so, how would you describe any hesitations?

17. How well would having the Vitals display work in the location/setting where you usually hold video visits with your care team?

17.1. Privacy issues due to use context?

17.2 Usability or connectivity issues due to use context?

17.3 Is a visual display sufficient for you or should there be an audio readout as well?

***Topic VII: Vitals RedCap Survey (Online) [10 mins]***

*Bring laptop with RedCap survey to Interviews. DO read aloud the introductory phrase for each section, then read each question, marking the patient’s response. Do NOT read aloud the Scale’s Name. Enter patient responses into the survey.*

Patient: <https://varedcap.rcp.vaec.va.gov/redcap/surveys/?s=MXPT8HCAFFPE7TXJ>

***Topic Area VIII: Conclusion and Participant characteristics [2 mins]***

To conclude, we would like to ask a few demographic questions.

18.1. What is the highest grade or level of school that you have completed?

- Did not complete high school
- High school graduate or GED
- Some college or 2-year degree
- 4-year college graduate or more

18.2. What is your race/ethnicity? You may select more than one.

- Black or African American (Examples: African American, Jamaican, Ethiopian, Haitian, etc.)
- American Indian or Alaska Native (Examples: Navajo Nation, Blackfeet Tribe, Mayan, Aztec, etc.)
- Asian (Examples: Chinese, Vietnamese, Indian, Lebanese, etc.)
- Native Hawaiian or Pacific Islander (Examples: Kanaka Maoli, Samoan, Chamorro, etc.)
- Hispanic, Latinx, or Spanish Origin (Examples: Puerto Rican, Cuban, Salvadoran, etc.)
- White (of European, Middle Eastern or North African descent, Examples: Irish, German, Italian, Lebanese, Arab, Moroccan or Caucasian)

18.3. What is your gender?

- Female
- Male
- Transgender
- Do not identify as male or female
- Decline to answer

Thank you for taking the time to share your experiences on Vitals with me today.

We appreciate your contribution to our project. [Turn off audio recorder]

**APPENDIX**

***All Potential Vital Signs to Prompt Participants [Only 6 Currently Available]***


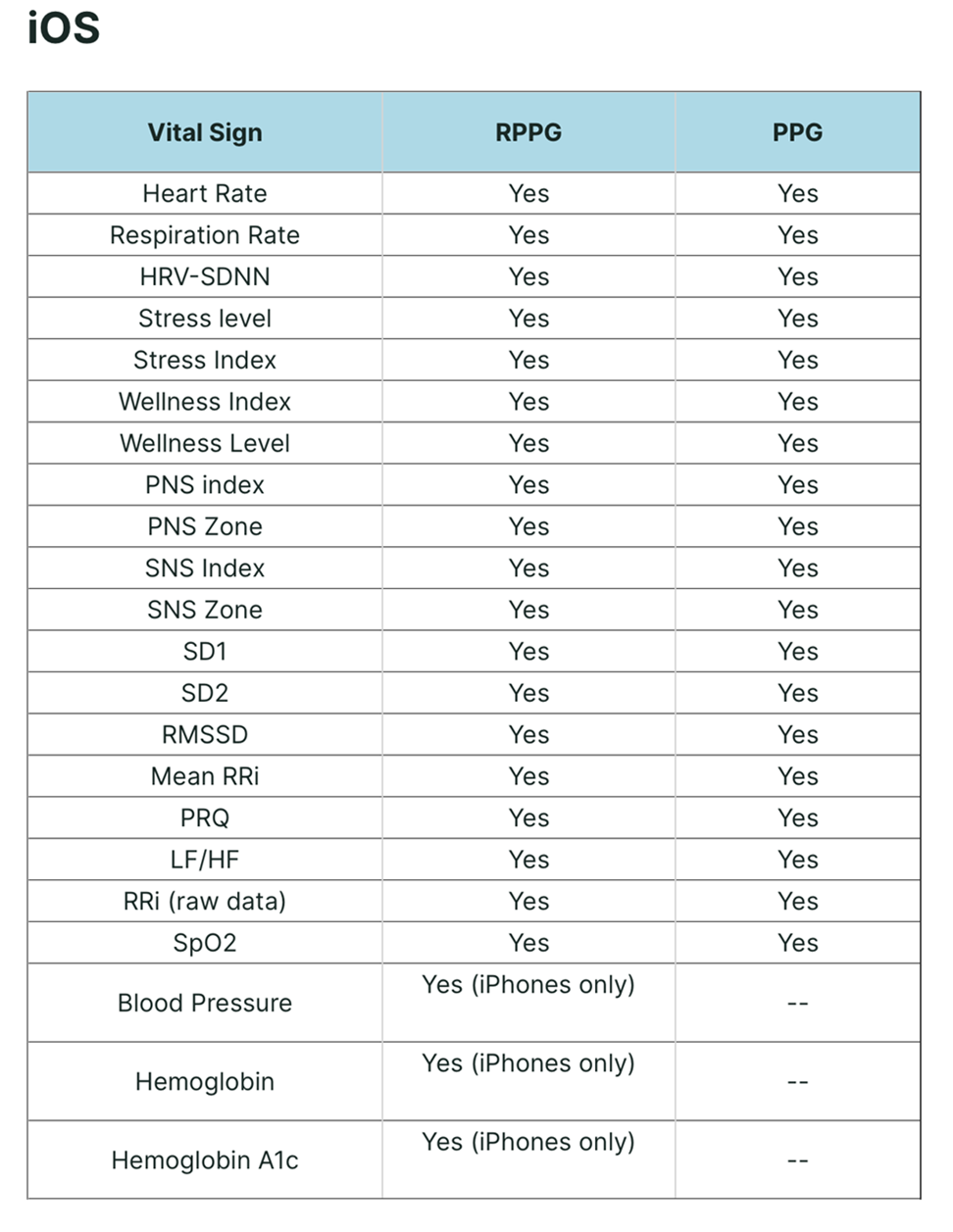


***Backup if Vitals Online is Not Operational: Think Aloud Procedure [3 mins]***

This screenshot shows what the Vitals reading looks like on your screen. Patients see a similar readout on their screens. Please note that the VVC screen in the demo may look a bit different from what you’re used to seeing.


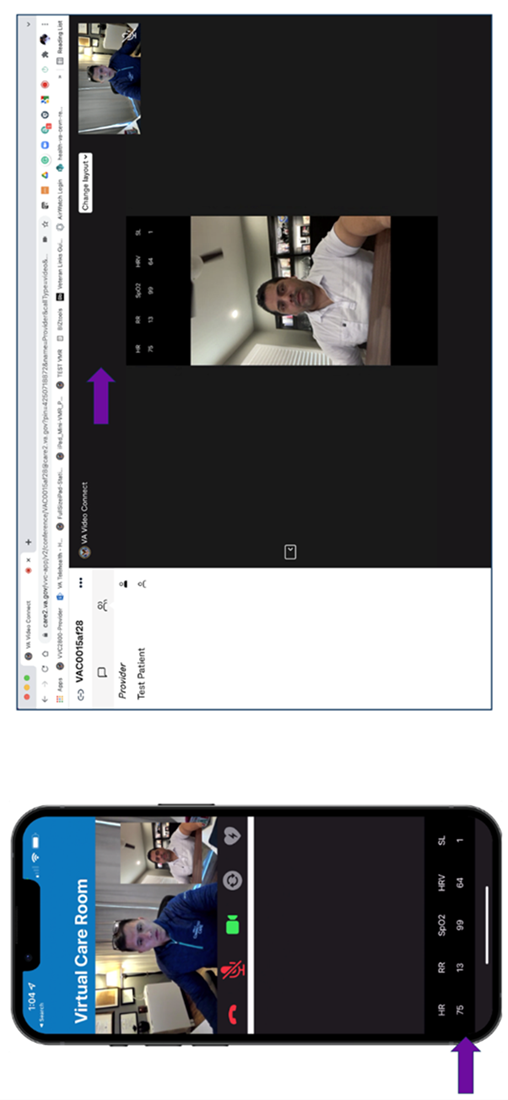

Supplement: Multimedia Appendix 2 [file formative_v8i1e60491_app2.docx]
