# Supplementary material for: Exploring Contactless Vital Signs Collection in Video Telehealth Visits Among Veterans Affairs Providers and Patients: Pilot Usability Study
Source: JMIR Form Res. 2024 Oct 23;8:e60491. doi: 10.2196/60491 (PMC11541150; doi:10.2196/60491)
Supplement: Multimedia Appendix 3 [file formative_v8i1e60491_app3.docx]

***VA Office of Connected Care Vitals Usability***

**(Provider version)**

|  | Completely disagree | Disagree | Neither agree nor disagree | Agree | Completely agree |
| --- | --- | --- | --- | --- | --- |
| 10-item Intervention Usability Scale (IUS) | | | | | |
| When thinking about your experience using the Vitals feature with your healthcare team… | | | | | |
| 1. I would use Vitals frequently. | ➀ | ➁ | ➂ | ➃ | ➄ |
| 2. I find Vitals unnecessarily complex. | ➀ | ➁ | ➂ | ➃ | ➄ |
| 3. I think Vitals is easy to use. | ➀ | ➁ | ➂ | ➃ | ➄ |
| 4. I would need the support of an  expert consultant to be able to  use Vitals. | ➀ | ➁ | ➂ | ➃ | ➄ |
| 5. I find the various components of Vitals are well integrated (that is, all parts of Vitals work well with the telehealth appointment). | ➀ | ➁ | ➂ | ➃ | ➄ |
| 6. I think there is too much inconsistency with Vitals (that is, it does not work well with the telehealth appointment). | ➀ | ➁ | ➂ | ➃ | ➄ |
| 7. I would imagine that most people would learn to use Vitals very quickly. | ➀ | ➁ | ➂ | ➃ | ➄ |
| 8. I find Vitals complicated to use. | ➀ | ➁ | ➂ | ➃ | ➄ |
| 9. I feel very confident (in my ability) using Vitals. | ➀ | ➁ | ➂ | ➃ | ➄ |
| 10. I would need to learn a lot of things before I could really use Vitals. | ➀ | ➁ | ➂ | ➃ | ➄ |
| Perceived Accuracy | | | | | |
| The accuracy of a feature like Vitals is based on its validity (measurement to a set standard) and its reliability (provides consistently valid measurement over time). Think of a thermometer – You want it to give you a valid reading of your body temperature (98^o^), and you want it to be reliable every time you use it. So with these two qualities in mind… | | | | | |
| 11. I think that Vitals is a valid instrument, that it measures to standard. | ➀ | ➁ | ➂ | ➃ | ➄ |
| 12. I think that Vitals is a reliable instrument, that validity of its readings will be consistent over time. | ➀ | ➁ | ➂ | ➃ | ➄ |
| 4-item Acceptability of Intervention Measure (AIM) | | | | | |
| After using vitals, think about your impression of using the Vitals feature with your healthcare team…. | | | | | |
| 13. Vitals meets my approval. (That is, I think Vitals is accurate and secure). | ➀ | ➁ | ➂ | ➃ | ➄ |
| 14. Vitals is appealing to me. (That is, saves time, easy to use, no pressure cuff) | ➀ | ➁ | ➂ | ➃ | ➄ |
| 15. I like Vitals. | ➀ | ➁ | ➂ | ➃ | ➄ |
| 16. I welcome Vitals. | ➀ | ➁ | ➂ | ➃ | ➄ |
| 4-item Intervention Appropriateness Measure (IAM) | | | | | |
| As tool to record my vital signs… | | | | | |
| 17. Vitals seems fitting. | ➀ | ➁ | ➂ | ➃ | ➄ |
| 18. Vitals seems suitable. | ➀ | ➁ | ➂ | ➃ | ➄ |
| 19. Vitals seems applicable. | ➀ | ➁ | ➂ | ➃ | ➄ |
| 20. Vitals seems like a good match. | ➀ | ➁ | ➂ | ➃ | ➄ |
| 4-item Feasibility of Intervention Measure (FIM) | | | | | |
| When completing a telehealth appointment with my healthcare team… | | | | | |
| 21. Vitals seems practical. | ➀ | ➁ | ➂ | ➃ | ➄ |
| 22. Vitals seems possible. | ➀ | ➁ | ➂ | ➃ | ➄ |
| 23. Vitals seems doable. | ➀ | ➁ | ➂ | ➃ | ➄ |
| 24. Vitals seems easy to use. | ➀ | ➁ | ➂ | ➃ | ➄ |
