# Supplementary material for: Exploring Contactless Vital Signs Collection in Video Telehealth Visits Among Veterans Affairs Providers and Patients: Pilot Usability Study
Source: JMIR Form Res. 2024 Oct 23;8:e60491. doi: 10.2196/60491 (PMC11541150; doi:10.2196/60491)
Supplement: Multimedia Appendix 5 [file formative_v8i1e60491_app5.docx]

**Revised Standards for Quality Improvement Reporting Excellence (SQUIRE 2.0) Checklist**

**Veterans Affairs Explores Contactless Vital Signs Collection**

**in Video Telehealth Visits: Usability Pilot Among Providers and Patients**

| **Text Section and Item Name** | **Section or Item Description** |
| --- | --- |
| **Title and Abstract** | |
| **1. Title** | **Indicate that the manuscript concerns an initiative to improve healthcare (broadly defined to include the quality, safety, effectiveness, patient-centeredness, timeliness, cost, efficiency, and equity of healthcare).**  The title “Veterans Affairs Explores Contactless Vital Signs Collection in Video Telehealth Visits: Usability Pilot Among Providers and Patients” reflects VA’s evaluation of a novel software feature called “Vitals.” Vitals allows patients to capture their own vital signs through the infrared camera on their smartphones to share with their VA healthcare providers, with the potential to improve the quality and efficiency of their healthcare. |
| **2. Abstract** | **a. Provide adequate information to aid in searching and indexing**  **b. Summarize all key information from various sections of the text using the abstract format of the intended publication or a structured summary such as: background, local problem, methods, interventions, results, conclusions.**  The abstract provides adequate information for search and indexing, following JMIR’s Abstract Format to summarize the study. |
| **Introduction** | *Why did you start?* |
| **3. Problem Description** | **Nature and significance of the local problem.**  Patient populations who face demographic, clinical, and geographic challenges (e.g., travel distance to care in rural settings) can benefit from video telehealth applications such as Vitals which offers remote contactless collection of vital signs through patients’ smartphones. However, this new technology required evaluation for usability among patients and providers before implementation within VA healthcare practice. |
| **4. Available knowledge**  **4. Available knowledge**  (continued) | **Summary of what is currently known about the problem, including relevant previous studies**  The Background section of this paper outlines the video telehealth advances by VA’s Office of Connected Health and Office of Rural Health to increase veteran’s access to care. If Vitals demonstrates accuracy in its readings (as tested through a separate project), it could enhance VA’s video-based care by providing accurate and automatic reporting and recording of vitals, sending patients’ vital readings (pending provider approval) directly to their electronic medical record; saving provider and patient time; and potentially reducing necessity of some home-based biometric devices. |

| **5. Rationale** | **Informal or formal frameworks, models, concepts, and/or theories used to explain the problem, any reasons or assumptions that were used to develop the intervention(s), and reasons why the intervention(s) was expected to work.**  In Methods – Step 1, we stated that usability testing included interviews with both healthcare providers and patients. The interview questions were informed by the constructs of the unified theory of acceptance and use of technology (UTAUT). UTAUT explores four elements of people’s willingness to adopt and use technology: perceived usefulness of the tool; ease of use; social influences, such as a physician’s recommendation; and facilitating conditions, such as digital training. |
| --- | --- |
| **6. Specific aims** | **Purpose of the project and of this report**  This study aimed to assess VA clinical provider and veteran patient attitudes regarding the usability of vitals. |
| **Methods** | *What did you do?* |
| **7. Context** | **Contextual elements considered important at the outset of introducing the intervention(s)**  To expand veterans’ access to health care, the Veterans Affairs (VA) Office of Connected Care explored a novel software feature called “Vitals” on its VA Video Connect telehealth platform. Vitals uses contactless video-based remote photoplethysmography (rPPG) through the infrared camera on veterans’ smartphones (and other devices) to automatically scan their faces to provide real-time vital statistics on screen to both the provider and patient. |
| **8. Intervention(s)**  **8. Intervention(s)**  (continued) | **a. Description of the intervention(s) in sufficient detail that others could reproduce it**  We conducted a mixed-methods evaluation of Vitals among VA providers and patients, collecting data in July and August 2023 at the VA Boston Healthcare System and VA San Diego Healthcare System. We conducted analyses in October 2023. In-person usability testing sessions consisted of 3 components: a think aloud procedure while using the software, a semi-structured interview and a 26-item web-based survey.  **b. Specifics of the team involved in the work**  The team included 3 PhD investigators from VA’s Center for Healthcare Optimization and Implementation Research in Boston, MA and the Deputy Director for Clinical Services/Telehealth Services of VA’s Office of Connected Care who is also a Staff Physician at VA San Diego, CA. |
| **9. Study of the Intervention(s)** | **a. Approach chosen for assessing the impact of the intervention(s)**  **b. Approach used to establish whether the observed outcomes were due to the intervention(s)**  In this mixed-methods evaluation of the Vitals software feature, in-person usability testing sessions with individual patients and healthcare providers consisted of: a think aloud procedure while using the software, a semi-structured interview and a 26-item web-based survey.  -The think aloud procedure provided observational notes by the study team based on the hands-on use of the Vitals app on smartphones and tablets in a mock video visit held with individual patient and provider participants.  -The semi-structured interview provided qualitative discussion quotes from patients and providers based on UTAUT constructs and related themes.  -The web-based survey completed by patients and providers offered quantitative scores on the usability, acceptability, feasibility and appropriateness of the Vitals software feature. |
| **10. Measures** | **a. Measures chosen for studying processes and outcomes of the intervention(s), including rationale for choosing them, their operational definitions, and their validity and reliability**  **b. Description of the approach to the ongoing assessment of contextual elements that contributed to the success, failure, efficiency, and cost**  **c. Methods employed for assessing data completeness and accuracy**  -The think aloud protocol did not apply measures. Participants unfiltered communication (verbal and non-verbal) on the usability and acceptability of the Vitals feature were observed or received and recorded by the team.  -The interview questions were informed by the 4 constructs of UTAUT theory: perceived usefulness of the tool; ease of use; social influences, such as a physician’s recommendation; and facilitating conditions, such as digital training.  -The 26-item survey quantitative survey that concluded each session was composed of 4 validated scales: the Intervention Usability Scale (IUS), the Acceptability of Intervention Measure (AIM), the Feasibility of Intervention Measure, and the Intervention Appropriateness Measure (IAM). All measures use Likert-style questions on a 5-point continuum. Based on research on the System Usability Scale (the parent scale for IUS), a score above 68 points is considered above average and anything below 68 points is below average. |
| **11. Analysis**  **11. Analysis**  **(continued)** | **a. Qualitative and quantitative methods used to draw inferences from the data**  **b. Methods for understanding variation within the data, including the effects of time as a variable**  -For qualitative analysis, audio recordings of usability testing sessions were transcribed. These, along with session notes, were coded thematically using rapid qualitative inquiry method. Researchers coded interviews, performing inductive and deductive content analysis. A priori categories included UTAUT constructs (performance expectancy, effort expectancy, social influence, and facilitating conditions) and other themes from the literature (Measurement and Instrument). Emergent themes from participants’ think aloud comments generated additional codes. The research team conducted a thematic analysis that assessed the patterns of attitudes and experiences and reached consensus on interpretation.  -For quantitative analysis, we calculated total scores for the survey measures following the published guidelines for scoring. For ease of interpretation, scores for the AIM, Feasibility of Intervention Measure, and IAM were converted to a 100-point scale to match the IUS scores. We then calculated descriptive statistics (e.g., range and mean) of the total scores on measures. We calculated descriptive statistics of individual items to capture some additional insights into participant responses. Analysis was conducted using R statistical software (version 4.2; R Foundation for Statistical Computing). |
| **12. Ethical Considerations** | **Ethical aspects of implementing and studying the intervention(s) and how they were addressed, including, but not limited to, formal ethics review and potential conflict(s) of interest**  The Institutional Review Board of the VA Boston Healthcare System determined this protocol [1685680-1] “VA Video Connect Vitals – Evaluation of Usability and Intent to Use” to be exempt on May 25, 2022.” |
| **Results** | *What did you find?* |
| **13. Results**  **13. Results**  (continued)  **13. Results**  (continued) | **a. Initial steps of the intervention(s) and their evolution over time (*e.g.*, time-line diagram, flow chart, or table), including modifications made to the intervention during the project**  **b. Details of the process measures and outcome**  **c. Contextual elements that interacted with the intervention(s)**  **d. Observed associations between outcomes, interventions, and relevant contextual elements**  **e. Unintended consequences such as unexpected benefits, problems, failures, or costs associated with the intervention(s).**  **f. Details about missing data**  We interviewed a representative sample of 20 VA providers and 13 patients at the VA Boston Healthcare System and VA San Diego Healthcare System.  **Usability of Vitals**  -Providers and patients highly rated Vitals’ usability (86 points and 82 points, respectively, on a 100-point scale). Per think aloud comments and interview quotes, providers found Vitals to be “useful” and “easy to use.”  - While Vitals usability scored high overall, providers rated 2 items on the IUS slightly lower, indicating the need for technical assistance at the introduction of the Vitals, and evidence of Vitals’ accuracy based on its validity (measurement to standard) and reliability (consistently valid measurement over time). During the structured interviews, providers also emphasized that educational materials distributed to both providers and patients before use would be critical to effective use.  - Patients did not express any concerns with usability of Vitals. However, several patients mentioned the challenge of logging in or maintaining an internet connection throughout the visit. Furthermore, some patients mentioned that it would be challenging to hold up their smartphone or tablet for the required 45-60 seconds (to obtain the vitals reading) and suggested that VA provide a tabletop stand to hold and stabilize the device during the reading.  **Acceptability of Vitals**  **-**Providers and patients rated Vitals high on the AIM reflecting their willingness to use Vitals. They perceived Vitals readings as being accurate, resulting in trust. Neither group expressed concern with security. Providers liked the fact that patients could take their own vitals anytime with the app, that it educates and engages them in their health, an important part of patient-centered care.  - Patients felt that Vitals was acceptable for their care stating that it helped them keep track of their vitals between visits, was easier than other ways of collecting vitals and helped with their telemedicine visits. They did not, however, want this to replace the option of seeing a doctor in-person.  **Feasibility of Vitals**  **-**In both, the surveys and interviews, providers and patients found Vitals highly feasible and practical for home-based use. One patient expressed that Vitals could also be empowering.  **Appropriateness of Vitals**  -Finally, providers and patients rated Vitals high on appropriateness, that is, fit for purpose, per the IAM measure. Providers believed that most clinical teams would readily integrate Vitals into their routine workflow because it saves time and delivers accurate, consistently collected vitals and may reduce reporting errors in the electronic health record.  -Furthermore, providers from different specialties indicated that Vitals is appropriate in a variety of clinical contexts. For example, several specialty care providers mentioned how Vitals would enhance their workflow, help with billing for video-based visits, and keep patients safer for preparation appointments. Specialty providers said Vitals could be used for video-based physical therapy appointments and medication management with the pharmacy. Also, primary care providers expressed that Vitals could help improve video-based visits and help streamline the collection of patient vitals. They also expressed interest in an application for follow-up visits. |
| **Discussion** | *What does it mean?* |
| **14. Summary** | **a. Key findings, including relevance to the rationale and specific aims**  **b. Particular strengths of the project**  While remote collection of vital readings has been described in the literature, this is one of the first accounts of testing a contactless vitals tool among providers and patients. If ongoing initiatives demonstrate accuracy in its readings, Vitals could enhance telemedicine by providing accurate and automatic reporting and recording of vitals, sending patients’ vital readings (pending provider approval) directly to their electronic medical record; saving provider and patient time; and potentially reducing necessity of some home-based biometric devices. Understanding these issues before US Food and Drug Administration approval of Vitals and its implementation could contribute to a seamless introduction of Vitals to VA providers and patients. |
